# Motor and Nonmotor Measures and Declining Daily Physical Activity in Older Adults

**DOI:** 10.1001/jamanetworkopen.2024.32033

**Published:** 2024-09-05

**Authors:** Shahram Oveisgharan, Tianhao Wang, Jeffrey M. Hausdorff, David A. Bennett, Aron S. Buchman

**Affiliations:** 1 Rush Alzheimer’s Disease Center, Rush University Medical Center, Chicago, Illinois; 2 Department of Neurological Sciences, Rush University Medical Center, Chicago, Illinois; 3 Department of Orthopedic Surgery, Rush University Medical Center, Chicago, Illinois; 4 Center for the Study of Movement, Cognition, and Mobility, Neurological Institute, Tel Aviv Sourasky Medical Center, Tel Aviv, Israel; 5 Department of Physical Therapy, Faculty of Medical and Health Sciences, and Sagol School of Neuroscience, Tel Aviv University, Tel Aviv, Israel

## Abstract

**Importance:**

Difficulties in identifying modifiable risk factors associated with daily physical activity may impede public health efforts to mitigate the adverse health outcomes of a sedentary lifestyle in an aging population.

**Objective:**

To test the hypothesis that adding baseline sensor-derived mobility metrics to diverse baseline motor and nonmotor variables accounts for the unexplained variance of declining daily physical activity among older adults.

**Design, Setting, and Participants:**

This cohort study analyzed data from participants of the Rush Memory and Aging Project (MAP), an ongoing longitudinal clinical pathological study that began to enroll older adults (age range, 59.4-104.9 years) in 1997. Wrist- and waist-worn sensors were added to MAP in 2005 and 2012, respectively, to record participants’ physical activity and mobility performances. Included participants were examined at baseline and annually followed up for a mean (SD) duration of 4.2 (1.6) years.

**Exposure:**

Twelve blocks of variables, including 3 blocks of mobility metrics derived from recordings of a belt-worn sensor to quantify a 32-foot walk, a Timed Up and Go (TUG) test, and a standing balance task, and 9 other blocks with 41 additional variables.

**Main Outcomes and Measures:**

A linear mixed-effects model was used to estimate the person-specific rate of change (slope) of total daily physical activity obtained from a wrist-worn sensor. Twelve linear regression models were used to estimate the adjusted *R*^2^ to quantify the associations of the variables with the slope.

**Results:**

A total of 650 older adults (500 females [76.9%]; mean [SD] age at baseline, 81.4 [7.5] years; 31 Black individuals [4.8%], 17 Latino individuals [2.6%], and 602 White individuals [92.6%]) were included. During follow-up, all but 1 participant showed declining daily physical activity, which was equivalent to approximately 16.8% decrease in activity level per year. In separate models, waist sensor–derived mobility metrics (32-foot walk: adjusted *R*^2^, 23.4% [95% CI, 17.3%-30.6%]; TUG test: adjusted *R*^2^, 22.8% [95% CI, 17.7%-30.1%]) and conventional motor variables (adjusted *R*^2^, 24.1% [95% CI, 17.7%-31.4%]) had the largest percentages of variance of declining daily physical activity compared with nonmotor variables. When the significant variables from all 12 blocks were included together in a single model, only turning speed (estimate [SE], 0.018 [0.006]; *P* = .005) and hand dexterity (estimate [SE], 0.091 [0.034]; *P* = .008) showed associations with declining daily physical activity.

**Conclusions and Relevance:**

Findings of this study suggest that sensor-derived mobility metrics and conventional motor variables compared with nonmotor measures explained most of the variance of declining daily physical activity. Further studies are needed to ascertain whether improving specific motor abilities, such as turning speed and hand dexterity, is effective in slowing the decline of daily physical activity in older adults.

## Key Points

**Question:**

Is the association of sensor-derived mobility metrics with declining daily physical activity greater in magnitude than that of conventional motor and nonmotor variables?

**Findings:**

In this cohort study of 650 older adults, sensor-derived mobility metrics and a conventional hand dexterity measure explained most of the variance of declining daily physical activity compared with conventional gait speed or nonmotor variables.

**Meaning:**

Findings of this study suggest the need for future studies to ascertain whether improving specific motor abilities, such as turning speed and hand dexterity, is effective in slowing the decline of daily physical activity in the aging population.

## Teaser

This cohort study assesses whether adding baseline sensor-derived mobility metrics to other baseline motor and nonmotor variables explains variance in daily physical activity decline among older adults.

## Introduction

Increasing daily physical activity and living a more active lifestyle are important^1^ to facilitate healthy aging via their diverse health benefits.^2^ Although trials have investigated methods of increasing daily physical activity in older adults,^3^ only half^4^ of (or even fewer^5^) older adults in related studies meet the recommended levels of daily physical activity.^1,6^ While causal inferences from observational studies are limited, these studies are crucial for identifying factors associated with daily physical activity that inform the design of physical activity intervention studies.^7^ Despite the recent advances in sensor technology and widespread availability of digital phenotyping of daily physical activity, even longitudinal studies of large numbers of older adults have identified a paucity of factors associated with declining daily physical activity.^8^

Mobility and the amount of daily physical activity are related but distinct phenotypes,^9^ underscoring the importance of examining both mobility and total daily physical activity. A wide variety of devices have been used to quantify different facets of mobility and physical activity. A previous study^10^ used waist-worn sensors to measure mobility and wrist-worn sensors to measure physical activity, as suggested by other studies,^11,12^ because movement measured at different locations is related to each other, and activity measures are related between the wrist, lower back, and ankle.^13^

In a recent longitudinal study examining diverse motor and nonmotor variables, we found that few variables were associated with declining daily physical activity, and they explained about only 20% of the variance of the slope of declining daily physical activity.^14^ An earlier cross-sectional study found that sensor-derived mobility metrics had a greater magnitude of association with daily physical activity compared with conventional motor performance and nonmotor variables.^10^ The current study was built on these prior studies and aimed to test the hypothesis that adding baseline sensor-derived mobility metrics to diverse baseline motor and nonmotor variables accounts for the unexplained variance of declining daily physical activity among older adults. In addition, to identify factors with the largest magnitude of association with the slope of declining physical activity, we calculated the effect size of the associations of different groups of variables to inform the design of future physical activity intervention studies.

## Methods

### Participants

Data were from participants enrolled in the Rush Memory and Aging Project (MAP), a longitudinal clinical pathological study of the chronic conditions of aging. Enrollment in MAP started in 1997 and included older adults (age range, 59.4-104.9 years) living in retirement centers and personal accommodations across northeastern Illinois. Inclusion criteria were no history of dementia and consent for annual clinical evaluations and for organ donation at death. The Rush University Medical Center Institutional Review Board approved the study. Written informed consent was obtained from the participants for annual testing. Details of MAP are provided elsewhere.^15^ We followed the Strengthening the Reporting of Observational Studies in Epidemiology (STROBE) reporting guideline.

From 1997 to 2023, 2258 participants were enrolled in MAP. Wrist sensors to record daily physical activity were deployed in 2005, and a belt-worn sensor for recording annual mobility testing was added in 2012. Therefore, the analytic baseline was the first MAP cycle at which a participant had both a valid recording of daily physical activity and valid recording of mobility tests. Moreover, to allow longitudinal analyses, participants were included only if they had 1 or more valid follow-ups of daily physical activity. Because of prior reports suggesting racial differences in motor function,^16^ we excluded 5 participants who did not self-identify as having Black, Latino, or White race and ethnicity; this exclusion yielded an analytic sample size of 650 (eFigure in Supplement 1). Comparison of included with excluded participants (eTable 1 in Supplement 1) showed that, on average, those excluded from analyses were older; had more dementia and disability; and reported less physical, cognitive, and social activity.

### Sensor-Derived Daily Physical Activity

A wrist sensor (Actical; Philips Healthcare) was used to obtain measures of total daily physical activity in the community setting. Participants were requested to wear this sensor on their nondominant wrist, which in turn recorded rest and activity continuously for 10 days. Details are provided in eMethods in Supplement 1 and elsewhere.^10^

We summed the activity counts of 15-second epochs for each 24-hour recording to yield daily physical activity counts, which were averaged across multiple days to construct the mean total physical activity counts per day.^17,18^ In prior work in this cohort, the intraclass correlation coefficient of daily physical activity counts was 0.82,^17,19^ supporting its use as a construct. A study using similar sensors reported estimated activity counts for common daily motor performances in older adults, including walking at 2.5 mph being associated with 2354 activity counts per minute.^13^

### Sensor-Derived Annual Mobility 

A sensor positioned on the waist (DynaPort MT; McRoberts) was worn by participants to record annual mobility testing. Details of the device and the procedures are provided in the eMethods in Supplement 1 and elsewhere.^10^

The 3 motor performances were the 32-foot walk, Timed Up and Go (TUG) test, and standing balance. In the 32-foot walk task, participants were instructed to walk at their own comfortable speed for a distance of 8 feet back and forth twice. In the TUG test, participants were asked to stand up from a chair, walk 8 feet forward, turn, walk 8 feet back, turn, and sit down in the same chair. To prevent duplication of similar walking measures in our analyses, we segmented the TUG test and included only 7 composite measures derived from 4 TUG subtasks, including both transitions (sitting to standing and standing to sitting) and turns. In the standing balance task, participants were asked to stand still for 20 seconds in 2 conditions: once with eyes closed, and once with eyes open.^20^ During the 32-foot walk and TUG performances, participants were allowed to use assistive walking devices.

Seventeen summary metrics grouped into 3 blocks corresponding to the 3 performances were used to quantify multiple facets of mobility (eTable 2 in Supplement 1).^20,21^ The mobility metrics have been examined in prior studies and were found to be related to health outcomes, supporting their use in the current study.^10,20,22^

In a subset of participants, research assistants also used a stopwatch and recorded the time that participants took to complete an 8-foot walk.^23^ The recorded time was used to estimate walking speed. Moreover, a composite variable was also calculated using a reciprocal of time and number of steps taken to walk 8 feet and to turn 360° twice.^24^

### Block Variables

Twelve blocks of variables were measured at baseline and examined in relation to longitudinal changes of daily physical activity, which was measured both at baseline and at follow-up periods. Block 1 included demographic variables, such as self-reported race and ethnicity. Blocks 2 to 4 included waist sensor–derived metrics of the 3 motor performances (32-foot walk, TUG test, and standing balance). Block 5 included other metrics from the wrist sensor–derived activity recording, such as variability of activity counts at baseline. Block 6 included vascular risk factors and diseases. Block 7 included different cognitive abilities, such as episodic memory and processing speed. Block 8 included psychosocial measures, such as depressive symptoms and purpose in life. Block 9 included pulmonary function measures, such as peak expiratory flow rate. Block 10 included self-reported late-life physical, cognitive, and social activities and disabilities. Block 11 included common motor performances, such as grip strength to assess motor function in older adults in both the research and clinical settings. Block 12 included laboratory measures, such as hemoglobin A_1c_ or estimated glomerular filtration rate. Details are provided in Table 1 and the eMethods in Supplement 1.

**Table 1.** Characteristics of Study Participants at Baseline (n = 650) and Their Associations With the Slope of Declining Daily Physical Activity^a^

Block of variables	Mean (SD)	Slope of declining daily physical activity^b^		
		Estimate (SE)^c^	FDR-corrected *P* value	Adjusted *R*^2^ (95% CI), %^d^
Demographics				14.8 (10.7-20.8)
Age, y	81.4 (7.5)	−0.0046 (0.0004)	<.001	14.3
Sex				
Female, No. (%)	500 (76.9)	−0.001 (0.008)	.88	0
Race and ethnicity, No. (%)^e^				
Black	31 (4.8)	−0.015 (0.016)	.44	0
Latino	17 (2.6)	−0.035 (0.021)	.18	0
White	602 (92.6)	1 [Reference]^e^		
Years of education completed	15.6 (3.0)	0.002 (0.001)	.18	1.2
Waist sensor–derived mobility metrics: 32-ft walk^f^				23.4 (17.3-3.6)
Regularity	0.09 (0.85)	0.022 (0.005)	<.001	16.7
Cadence	0.12 (0.95)	0.008 (0.004)	.04	7.9
Pace	0.18 (1.02)	0.027 (0.004)	<.001	19.8
Variability of step time	−0.13 (0.87)	0.007 (0.004)	.10	2.6
Waist sensor–derived mobility metrics: TUG test^f^				22.8 (17.7-30.1)
Stand up complexity	−0.12 (0.66)	0.001 (0.008)	.86	7.6
Stand up duration	−0.14 (0.85)	−0.010 (0.006)	.28	10.8
Sit down control	0.07 (0.85)	−0.005 (0.005)	.67	5.3
Sit down smoothness	−0.32 (0.82)	−0.002 (0.005)	.86	0.8
Turning speed	0.06 (0.93)	0.040 (0.005)	<.001	22.9
Duration of first turn	−0.06 (1.01)	-.001 (0.005)	.86	10.2
Duration of the second turn	0.01 (1.02)	−0.004 (0.005)	.74	7.7
Waist sensor–derived mobility metrics: standing balance^f^				3.1 (1.3-7.8)
Eyes open				
Sway magnitude	0.17 (0.96)	−0.021 (0.010)	.12	2.0
Sway frequency	0.08 (1.02)	−0.013 (0.007)	.17	0.4
Sway jerk	0.16 (1.01)	0.013 (0.011)	.26	2.6
Eyes closed				
Sway magnitude	0.46 (0.95)	0.015 (0.010)	.22	1.0
Sway frequency	0.57 (1.07)	0.007 (0.007)	.32	0.6
Sway jerk	0.77 (1.10)	−0.024 (0.010)	.11	2.5
Wrist sensor–derived: other variables^f^				12.0 (7.5-18.7)
Interdaily stability	0.50 (0.12)	0.065 (0.030)	.08	1.3
Intradaily variability	0.72 (0.16)	−0.218 (0.055)	<.001	11.8
Fractal alpha1	0.92 (0.06)	−0.121 (0.153)	.70	7.4
Fractal alpha2	0.82 (0.09)	0.026 (0.045)	.70	2.1
Sleep fragmentation	0.03 (0.01)	0.002 (0.631)	<.001	0
Vascular factors				4.6 (1.8-9.2)
No. of vascular risk factors (0-3), median (IQR)^g^	1.0 (1.0 – 2.0)	−0.006 (0.005)	.23	0.7
No. of vascular diseases (0-4), median (IQR)^h^	0.0 (0.0 – 1.0)	−0.028 (0.005)	<.001	4.6
BMI	27.1 (5.2)	−0.0001 (0.0007)	.23	0.3
Cognition^f^				11.0 (6.9-17.3)
Episodic memory	0.23 (0.73)	0.007 (0.006)	.39	4.9
Processing speed	0.14 (0.74)	0.029 (0.006)	<.001	9.4
Semantic memory	0.18 (0.68)	0.002 (0.007)	.81	5.9
Working memory	0.07 (0.75)	−0.001 (0.005)	.81	3.0
Visuospatial ability	0.14 (0.77)	0.015 (0.005)	.005	5.9
Psychosocial				7.7 (4.3-13.1)
Depressive symptoms (0-10)	0.83 (1.40)	−0.003 (0.003)	.53	1.9
Purpose in life (1-5)	3.78 (0.47)	0.054 (0.010)	<.001	7.4
Social network size	7.37 (5.47)	0.0002 (0.0007)	.81	0
Loneliness (1-5)	2.12 (0.55)	0.008 (0.008)	.53	1.2
Well-being (1-7)	5.59 (0.56)	0.003 (0.009)	.81	3.1
Pulmonary function^i^				5.4 (2.3-9.9)
FEV_1_, mean (SD), L	1.66 (0.54)	0.006 (0.010)	.55	3.7
FVC, L	1.87 (0.59)	0.006 (0.008)	.51	3.0
Peak expiratory flow rate, L/min	288.0 (104.1)	0.0002 (0.00003)	<.001	5.4
Self-reported activities and disabilities				19.8 (14.1-29.2)
Physical exercise, h/wk	3.57 (3.50)	0.0030 (0.0009)	.003	4.2
Cognitive activity (1-5)	3.18 (0.64)	−0.005 (0.005)	.44	0.6
Social activity (1-5)	2.68 (0.55)	0.019 (0.006)	.004	6.0
Disability in IADL (0-8)	0.89 (1.42)	−0.015 (0.003)	<.001	15.4
Disability in basic ADL (0-6)	0.16 (0.58)	0.0009 (0.0065)	.89	4.5
Mobility disability (0-3)	0.67 (0.93)	−0.017 (0.004)	<.001	14.0
Conventional motor variables^f^				24.1 (17.7-31.4)
Hand strength	1.08 (0.29)	0.021 (0.012)	.09	6.9
Hand dexterity	1.03 (0.16)	0.120 (0.029)	<.001	20.2
Square root of Parkinsonism severity score	1.86 (1.34)	−0.021 (0.003)	<.001	20.9
Laboratory				6.0 (3.6-11.1)
HbA_1c_, %	5.82 (0.57)	−0.010 (0.007)	.19	0.6
LDL-C, mg/dL	98.2 (32.2)	0.000 (0.000)	.96	0.4
HDL-C, mg/dL	64.1 (18.7)	0.0007 (0.0002)	.004	1.1
Triglyceride, mg/dL	125.4 (57.9)	0.0001 (0.0001)	.10	0
Thyrotropin (previously thyroid-stimulating hormone), mIU/L	2.50 (2.40)	−0.0009 (0.0015)	.63	0
Hemoglobin, g/dL	13.4 (1.2)	0.0094 (0.0029)	.004	1.9
eGFR, mL/min/1.73m^2^	67.8 (17.2)	0.0008 (0.0002)	<.001	3.5

Abbreviations: ADL, activities of daily living; BMI, body mass index (calculated as weight in kilograms divided by height in meters squared); eGFR, estimated glomerular filtration rate; FDR, false discovery rate; FEV_1_, forced expiratory volume in the first second of expiration; FVC, forced vital capacity; HbA_1c_, hemoglobin A_1c_; HDL-C, high-density lipoprotein cholesterol; IADL, instrumental activity of daily living; LDL-C, low-density lipoprotein cholesterol; TUG, Timed Up and Go test.

SI conversion factor: To convert eGFR to milliliter per second per square meter, multiply by 0.0167; HbA_1c_ to proportion of total hemoglobin, multiply by 0.01; HDL-C and LDL-C to millimoles per liter, multiply by 0.0259; hemoglobin to gram per liter, multiply by 10.0; triglycerides to millimoles per liter, multiply by 0.0113.

^a^ In 12 separate linear regression models, each of the 12 blocks of variables with the slope of declining daily physical activity was examined.

^b^ The estimate (SE), FDR-corrected *P* value, and adjusted *R*^2^ of each block were calculated with all included variables in the model, while the adjusted *R*^2^ of each variable was calculated by including the examined variable in a single model.

^c^ To interpret the estimates, the activity counts were divided by 10^5^ and log-transformed. For example, the age estimate was −0.0046. Therefore, the slope of decline for an individual 10 years older than the average participant (91-year-old vs 81-year-old participants) was 10 × −0.0046 = −0.046 faster than the mean rate of decline, which was −0.184. It means that the 91-year-old participant had exp(0.184 + 0.046) = 1.26 × 10^5^ lower daily activity count per year. In comparison, the 81-year-old participant had exp(0.184) = 1.20 × 10^5^ lower daily activity count per year. The difference (6000 activity count per year faster declining activity count in the 91-year-old participant) was equal to approximately 3 minutes of walking at 2.5 mph^13^.

^d^ The 95% CI was calculated for the blocks of variables, rather than the variables per se, because the blocks were the primary interest of the study.

^e^ Race and ethnicity data were self-reported by participants and were examined using 2 dummy variables indicating Black and Latino compared with White as the reference level.

^g^ Number of vascular risk factors indicates number of hypertension, diabetes, and smoking present in a participant.

^h^ Number of vascular diseases indicates number of stroke, myocardial infarction, congestive heart failure, and lower extremities claudication present in a participant.

^i^ The FEV_1_ and FVC had multicollinearity (variance inflation factors were 19.0 and 14.2). Therefore, they were examined in separate models, each also included peak expiratory flow. However, the same 5% variances were explained by the 2 models and a model that included only the peak expiratory flow as the model term. Therefore, peak expiratory flow rate was the variable examined with other significant variables in a single model.

### Statistical Analysis

The eMethods in Supplement 1 provide details about the statistical analyses. Using a linear mixed-effects model, we estimated person-specific slope of change in daily physical activity. We transformed physical activity counts by a natural logarithmic transformation to meet assumptions of linear mixed-effects models.^14,25^ In 12 linear regression models, we examined the associations of variables, classified in 12 blocks, with the person-specific slope of change in daily physical activity. We used the adjusted *R*^2^ for each of the 12 linear regression models to quantify the percentage of variance of the slope of change of daily physical activity that was explained by each of the 12 blocks of variables. We used bootstrapping with 1000 repetitions to obtain 95% CIs of the adjusted *R*^2^. In a final, single model, we examined all of the variables associated with the slope of change of daily physical activity in the individual blocks of variables. Multicollinearity was present if variance inflation factor was greater than 10.

To address multiple comparisons, we applied the Benjamini and Hochberg false discovery rate (FDR).^26^ The FDR was applied separately in each block, including the 3 blocks of sensor-derived metrics of the 3 motor performances. Statistical significance was indicated by FDR-corrected *P* < .05. Analyses were conducted between February 2023 and June 2024 using SAS version 15.2 (SAS Institute Inc).

## Results

Baseline characteristics of the 650 participants in the study are summarized in Table 1. These participants included 500 females (76.9%) and 150 males (23.1%); had a mean (SD) age at baseline of 81.4 (7.5) years; and self-identified as being of Black (31 [4.8%]), Latino (17 [2.6%]), or White (602 [92.6%]) race and ethnicity.

**Figure 1.**
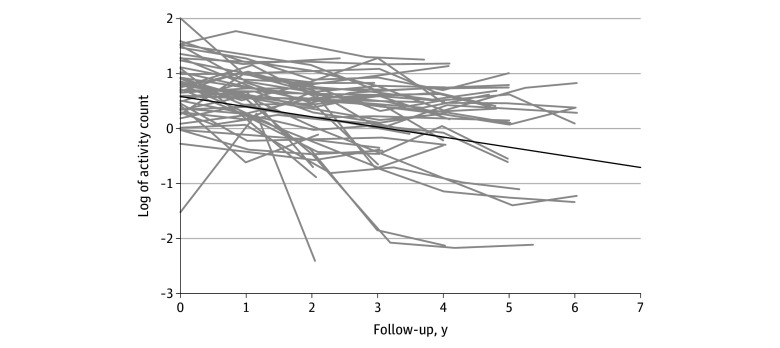
Trajectories of Declining Daily Physical Activity of 50 Randomly Selected Participants The gray lines illustrate person-specific trajectories of daily physical activity of 50 randomly selected participants. The superimposed black line illustrates the mean trajectory of declining daily physical activity estimated by a mixed-effects model, including all 650 participants.

During a mean (SD) follow-up of 4.2 (1.6) years, all but 1 participant showed a negative estimated slope of change in daily physical activity, indicating that all but 1 participant showed declining daily physical activity (Figure 1). The estimate (SE) of slope of decline was −0.184 (0.007) unit per year in the log-transformed scale, which was equivalent to approximately 16.8% per year reduction in the level of physical activity (eTable 3 in Supplement 1).

**Figure 2.**
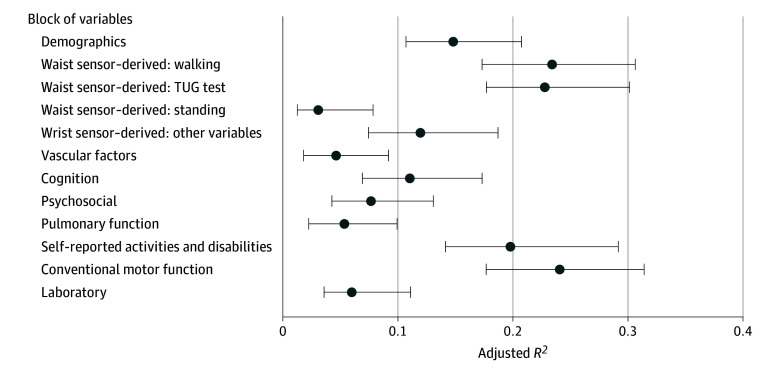
Variance of the Slope of Physical Activity Decline by Different Blocks of Variables Each circle and its whisker illustrates the percentage of variance and 95% CI of slopes of declining daily physical activity accounted for by a block of variables. To obtain the percentages, 12 separate linear regression models were examined, with slope of declining daily physical activity as the outcome and 1 block of variables as the model term. The model’s adjusted *R*^2^ provided the percentage of variance of the slope of declining daily physical activity accounted for by the block of variables. To obtain the 95% CIs, bootstraping with 1000 repetitions was performed. TUG indicates Timed Up and Go.

When examining the 12 blocks of variables in associations with the person-specific slopes of declining daily physical activity (Figure 2), we found that the largest percentages of variance of declining daily physical activity were accounted for by conventional motor variables (adjusted *R*^2^, 24.1% [95% CI, 17.7%-31.4%]) and waist sensor–derived mobility metrics (32-foot walk: adjusted *R*^2^, 23.4% [95% CI, 17.3%-30.6%]; TUG test: adjusted *R*^2^, 22.8% [95% CI, 17.7%-30.1%]) (Table 1). Blocks of nonmotor measures, including psychosocial variables and other health variables, accounted for less than 10% of the variance of declining daily physical activity.

The most widely available metric used to assess gait in older adults was gait speed.^27^ We obtained gait speed using a stopwatch in 323 participants (49.7%), with a mean (SD) of 0.51 (0.17) m/s. Slower gait speed was associated with a faster decline in daily physical activity (estimate [SE], 0.181 [0.027]; *P* < .001). However, gait speed alone explained only 11.7% (95% CI, 5.2%-19.3%) of the variance of declining daily physical activity. In contrast, the sensor-derived walking variables in the same participants accounted for 22.9% (95% CI, 13.1%-34.1%) of the variance of declining daily physical activity (eTable 4 in Supplement 1). When we replaced gait speed with a summary variable derived from the time and number of steps taken to complete an 8-foot walk and 360° turn,^24^ the explained variance of declining daily physical activity was improved to 20.1% (95% CI, 10.7%-30.4%), which highlighted the superiority of more sophisticated measures of gait rather than gait speed as a proxy for health.

**Figure 3.**
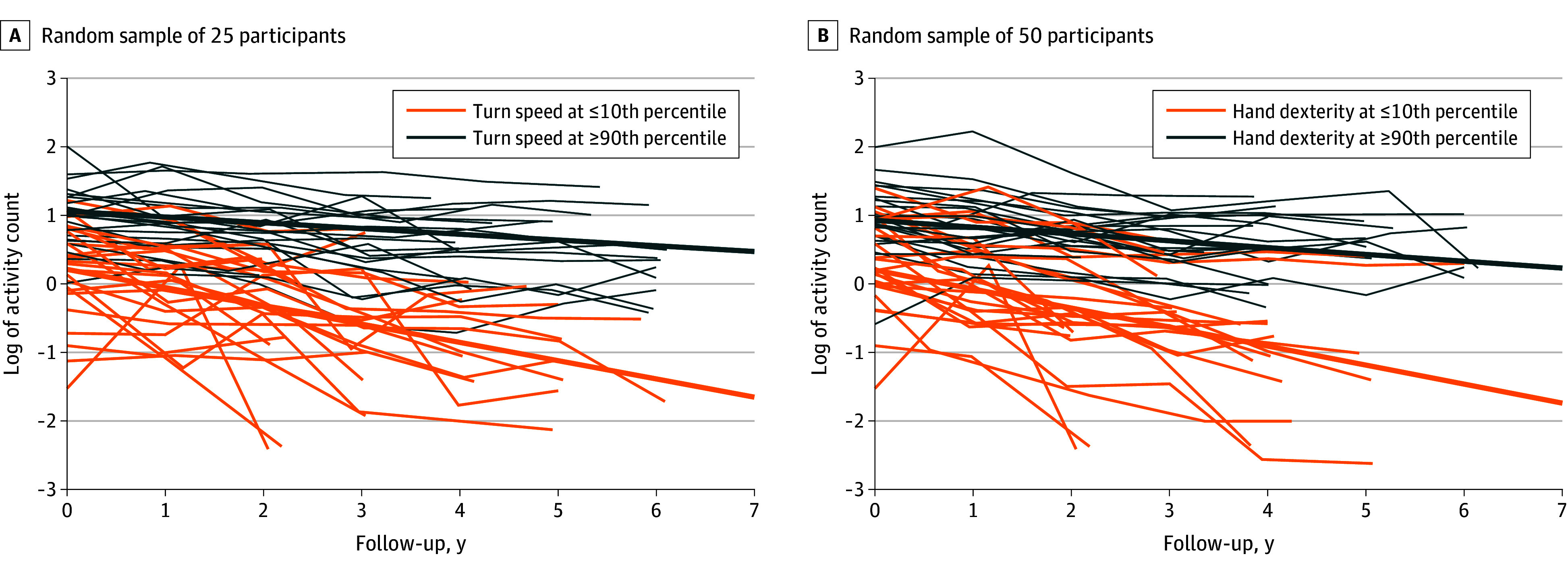
Trajectories of Physical Activity in Participants With Different Levels of Turning Speed and Hand Dexterity Levels at or greater than 90th percentile indicate high levels, and levels at or below than 10th percentile are low levels. Blue bold lines illustrate the model-estimated mean trajectories of physical activity for the 2 levels of turning speed and hand dexterity.

In a single model, we examined all variables that were associated with declining daily physical activity from each of the 12 blocks. In this single model that included 20 variables (Table 2), only sensor-derived turning speed (estimate [SE], 0.018 [0.006]; *P* = .005) and hand dexterity (estimate [SE], 0.091 [0.034]; *P* = .008) remained associated with declining daily physical activity (Figure 3). The variance of declining daily physical activity explained by this model was 29.8% (95% CI, 25.9%-40.4%), which was 50% more than in a prior study that did not include sensor-derived mobility metrics and explained 21% of the variance of daily physical activity.

**Table 2.** Associations of Variables With Declining Daily Physical Activity in a Single Model^a^

Block of variables	Declining daily physical activity rate	
	Estimate (SE)	*P* value
Demographics		
Age at baseline, y	−0.001 (0.001)	.43
Waist sensor–derived mobility metrics: walking		
Regularity	0.003 (0.006)	.63
Cadence	−0.005 (0.004)	.21
Pace	0.007 (0.005)	.19
Waist sensor–derived mobility metrics: TUG test		
Turning speed	0.018 (0.006)	.005
Wrist sensor–derived: other variables		
Intradaily variability	−0.038 (0.025)	.13
Vascular factors		
No. of vascular diseases present	0.006 (0.006)	.28
Cognition		
Processing speed	−0.003 (0.006)	.57
Visuospatial ability	0.005 (0.005)	.30
Psychosocial		
Purpose in life	0.008 (0.009)	.35
Pulmonary function		
Peak expiratory flow	0.0000 (0.0000)	.80
Self-reported activities and disabilities		
Physical exercise	0.001 (0.001)	.26
Social activity	0.004 (0.007)	.61
IADL	−0.005 (0.004)	.17
Mobility disability	−0.0002 (0.0052)	.97
Conventional motor variables		
Hand dexterity	0.091 (0.034)	.008
Square root of Parkinsonism severity score	−0.004 (0.004)	.27
Laboratory		
HDL-C	0.0003 (0.0002)	.17
Hemoglobin	0.004 (0.003)	.21
GFR	0.0002 (0.0002)	.26

Abbreviations: GFR, glomerular filtration rate; HDL-C, high-density lipoprotein cholesterol; IADL, instrumental activity of daily living; TUG, Timed Up and Go test.

^a^ In a single linear regression model, variables (n = 20) associated with declining daily physical activity when examined in their blocks were examined together in a single model with slopes of declining daily physical activity as the outcome. Model-derived estimates (SEs) and *P* values are shown.

### Sensitivity Analysis

Participants with dementia had the fastest slope of daily physical activity decline followed by participants with mild cognitive impairment and no cognitive impairment (mean [SD], −0.27 [0.09]; −0.22 [0.09]; −0.17 [0.09], respectively), which were equal to 23.7%, 19.7%, and 15.6% lower levels of daily physical activity counts per year, respectively (*F* = 22.9; *P* < .001). In a sensitivity analysis, we excluded participants with dementia and repeated the main analyses. This analysis of adults without dementia indicated that blocks including motor variables explained most of the variance (>20%) of declining daily physical activity (eTable 5 in Supplement 1).

During the baseline walking and TUG tests, 23 of 650 participants (3.5%) had used a walker, 10 (1.5%) a cane, and 2 (0.3%) other devices. Use of device at baseline was associated with faster decline in daily physical activity (mean [SD], −0.26 [0.13] vs −0.18 [0.09]; *t* = 3.6; *P* < .001). However, exclusion of participants who had used walking aids did not change the main finding that motor variables explained most of the variance of declining daily physical activity (eTable 6 in Supplement 1). In further sensitivity analyses, exclusion of participants with a diagnosis of Parkinson disease (n = 21; eTable 7 in Supplement 1) or those who had died during the study (n = 242; eTable 8 in Supplement 1) did not change the main finding that sensor-derived mobility metrics and conventional motor variables explained most of the variance of declining daily physical activity.

The main outcome, the slope of declining physical activity, was estimated using a linear mixed-effects model. In a sensitivity analysis, we estimated each participant’s slope of declining physical activity using 650 separate linear regression models and examined the 12 blocks of variables in relation to the slopes. The analysis did not change the main finding that motor variables explained most of the variance of the slope of declining physical activity (32-feet walk: 6.4%; TUG test: 5.9%) (eTable 9 in Supplement 1). However, the maximum (6.4%) explained variance of the linear regression–based slope of declining physical activity was much smaller than the explained variance of the slope estimated by the linear mixed-effects model because the slopes of the linear regressions were estimated without borrowing information of between-participants’ variation and were noisier. In further analyses, we did not estimate the slopes using statistical models and simply calculated the difference in the levels of physical activity at baseline and at first follow-up divided by the time interval between the visits. Using this calculated change in daily physical activity as the outcome did not change the study finding that motor variables (conventional motor measures, 3.6%) explained most of the variance of declining daily physical activity (eTable 10 in Supplement 1).

In 157 participants with 6 or more measurements of physical activity, we examined the associations of the most promising factors of declining physical activity, turning speed, and hand dexterity at baseline with the level of physical activity at years 2, 4, and 6. The models were controlled for age, sex, physical activity at baseline, and the time interval between baseline and year of follow-up assessment. The analyses indicated that the associations of turning speed and hand dexterity with physical activity assessed years later were not attenuated by longer duration of follow-up; for example, the association with physical activity at year 6, had an estimate (SE) of 0.222 (0.059; *P* < .001) for turning speed and 1.109 (0.382; *P* = .004) for hand dexterity (eTable 11 in Supplement 1).

## Discussion

This study of community-dwelling older adults used 2 different sensors to quantify both mobility and total daily physical activity. During 4 years of follow-up, daily physical activity declined in almost all participants. This finding highlights the public health importance of identifying factors associated with daily physical activity that may be modifiable. Adding sensor-derived mobility metrics to a wide range of conventional motor and nonmotor variables increased the explained variance of declining daily physical activity by approximately 50% compared with a prior study that used no sensor metrics.^14^ While daily physical activity may be affected by diverse factors, in a single model including both motor and nonmotor variables, only sensor-derived mobility metrics and conventional motor variables remained associated with declining daily physical activity. These results support more detailed characterization of mobility using sensors that can complement conventional motor variables and may provide information on targeted mobility interventions to maintain daily physical activity in an aging population.

Unobtrusive sensors provide quantitative metrics of both exercise and habitual physical activity, which may represent a larger percentage of daily physical activity in aging adults. These sensors circumvent the limitations of traditional self-report questionnaires of physical activity that may overestimate daily physical activity.^28,29^ Prior studies in this cohort showed that a sensor-derived measure of physical activity had a greater magnitude of association with incident cognitive^30^ and motor^31^ impairment than self-reported measures of physical activity, which was also supported by other studies.^32^

Walking is a complex behavior that requires the production of coordinated rhythmic patterns of muscle activation of both the lower and upper extremities. Hence, it is not surprising that studies of walking have placed sensors on the arm, trunk, and/or ankles^11,33^ and that there are associations of activity measured at each site.^34^ Body locations around the lower trunk are deemed as the best locations for measurement of activity counts, but most studies of older adults have mounted sensors on the wrists^11^ because such placement is less intrusive and a more acceptable option. Studies have suggested that activity counts derived from the nondominant wrist are more reflective of the counts obtained by a trunk sensor.^35^ Similarly, although the ankle is the recommended location for quantifying gait, older adults prefer waist over ankle because waist is a more familiar location to wear accessories.^12,36^ Moreover, many waist-worn sensor-derived gait metrics had high rates of concordance with ankle-derived metrics.^12^ Therefore, prior studies support the body locations where sensors were mounted in the current study.

Decades of research have shown that gait speed is a robust but nonspecific factor associated with incident adverse health outcomes in older adults.^37^ In the current study, addition of sensor-derived mobility metrics to conventional motor and nonmotor variables increased the explained variance of declining physical activity by 50%, compared with a previous study,^14^ a finding consistent with other studies that suggested an assessment of a wider array of sensor-derived gait and balance measures is needed to describe the varied facets of mobility not captured by simply measuring gait speed. Standardization of devices and extracted metrics as well as an equivalent clinical lexicon for varied impairment of the metrics are crucial to translating these digital advances from research to the clinical setting for the assessment and clinical care of impaired mobility in older adults.

The finding that the motor function–related variables had a great magnitude of association with slower decline in daily physical activity may provide information on interventions to promote levels of physical activity in older adults. Several trials have examined different methods for increasing the levels of physical activity in older adults,^38^ with most of them targeting participants’ motivation in pursuing a more active lifestyle.^38^ However, a meta-analysis of the results of these trials showed small benefits during short-term follow-up and no long-term outcomes, which can be explained by the small effect size of the association between psychosocial factors and declining daily physical activity in the current study. These findings suggest that interventions should focus on improving motor abilities.

When examined in separate blocks, sensor-derived gait metrics indicative of slower walking speed, more irregular steps, and shorter strides at baseline were associated with faster declines in physical activity. However, in the final stage of this analysis, only the sensor-derived turning speed from the TUG test and hand dexterity remained associated with declining daily physical activity. Slower turning speed has been reported as a risk factor for varied adverse health outcomes.^20,39,40^ It has been suggested that more cortical control may be required during turning vs walking straight, which makes slower turning speed a more sensitive proxy for impaired neural system, which is required for planning and accomplishing physical activity. One study suggested that a targeted intervention, a home-based exercise program, can increase turning speed.^41^ It is unclear, however, if this improvement would be reflected in daily physical activity.

It is unclear why hand dexterity was associated with declining daily physical activity. It is possible that hand dexterity is a proxy for motor speed. Another explanation is that the eye-hand coordination for successful peg placement is a marker of a motor ability crucial for more daily physical activity. Hand dexterity may also serve as a proxy for other functions that are important for physical activity, including executive cognitive functioning.^42^

### Limitations

The study has several limitations. The data analyzed were from an observational study; thus, drawing causal inferences will require further replication in prospective intervention studies. Although a bidirectional relationship may exist between motor abilities and physical activity, the results showed no attenuation in the association between baseline mobility and repeated longitudinal measures of daily physical activity despite longer duration of follow-up. These results suggest a longitudinal rather than a cross-sectional association between better mobility at baseline and higher levels of physical activity during follow-up assessments. The primary outcome measure (slope of declining physical activity) was estimated using a linear mixed-effects model, which might have not been an accurate measure of longitudinal change in physical activity, although using alternative approaches yielded similar findings. Most participants were White persons with more years of education than the general population. Additionally, while prior studies had suggested racial differences in motor function,^16^ this study did not find any association between race and ethnicity and slope of declining physical activity, which might be in part due to inadequate power given that 92.6% of the analytic sample were White participants. Hence, the findings will need to be replicated in more diverse cohorts.

## Conclusions

In this cohort study, daily physical activity declined in almost all participants. Sensor-derived mobility metrics and conventional motor variables showed the largest percentages of variance of the decline in physical activity compared with nonmotor variables. These findings support the importance of measuring a wide array of sensor-derived gait and balance variables to more fully describe the varied facets of mobility that are not captured by measuring gait speed alone. Yet, further studies will be needed before sensors can be deployed routinely in diverse clinical setting for the assessment of older adults. Physical exercise interventions to improve specific motor abilities, such as turning speed and hand dexterity, must be examined in prospective clinical trials to test their effectiveness in slowing the decline of physical activity in older adults.

## Notes

Supplement 1. **eFigure.** Flow Chart of Sample Selection From the Rush Memory and Aging Project (MAP) Participants **eTable 1.** Comparison of Included vs Excluded Participants **eTable 2.** The 17 Summary Metrics Derived From the Waist Sensor Recordings Quantifying Three Performances: A 32 ft Walk, a Timed Up and Go (TUG) Task, and Standing Balance Tasks eMethods **eTable 3.** A Linear Mixed Effects Model to Estimate Person-Specific Slope of Change of Daily Physical Activity **eTable 4.** Associations of Sensor-Derived Walking Metrics With the Rate of Physical Activity Decline in Participants With Valid Manually Calculated Gait Speed (n=323) **eTable 5.** Variance of Physical Activity Decline Rate Explained by Different Blocks of Covariates in Participants Without Dementia (n=630) **eTable 6.** Variance of Physical Activity Decline Rate Explained by Different Blocks of Covariates in Participants Who Did Not Use Walking Aid During Testing (n=615) **eTable 7.** Variance of Declining Daily Physical Activity Explained by Different Blocks of Covariates in Participants Who Did Not Have a Parkinson’s Disease Diagnosis (n=629) **eTable 8.** Variance of Declining Daily Physical Activity Explained by Different Blocks of Covariates in Participants Who Did Not Die During the Follow Up (n=408) **eTable 9.** Use of the Slope of Declining Daily Physical Activity Estimated by 650 Separate Linear Regression Models for Identification of the Variance of Declining Daily Physical Activity Explained by Different Blocks of Covariates **eTable 10.** Use of the Change in the Level of Physical Activity Between Baseline and the First Follow Up Divided by the Visits’ Time Interval as the Outcome in Relation to the 12 Blocks of Variables **eTable 11.** Associations of Baseline Turn Speed and Hand Dexterity With the Level of Physical Activity at Years 2, 4, and 6 in 157 Participants Who Had 6+ Measurements of Physical Activity

Supplement 2.
Data Sharing Statement
