# Supplementary material for: Motor and Nonmotor Measures and Declining Daily Physical Activity in Older Adults
Source: JAMA Netw Open. 2024 Sep 5;7(9):e2432033. doi: 10.1001/jamanetworkopen.2024.32033 (PMC11378007; doi:10.1001/jamanetworkopen.2024.32033)
Supplement: Supplement 1. — eFigure. Flow Chart of Sample Selection From the Rush Memory and Aging Project (MAP) Participants eTable 1. Comparison of Included vs Excluded Participants eTable 2. The 17 Summary Metrics Derived From the Waist Sensor Recordings Quantifying Three Performances: A 32 ft Walk, a Timed Up and Go (TUG) Task, and Standing Balance Tasks eMethods eTable 3. A Linear Mixed Effects Model to Estimate Person-Specific Slope of Change of Daily Physical Activity eTable 4. Associations of Sensor-Derived Walking Metrics With the Rate of Physical Activity Decline in Participants With Valid Manually Calculated Gait Speed (n=323) eTable 5. Variance of Physical Activity Decline Rate Explained by Different Blocks of Covariates in Participants Without Dementia (n=630) eTable 6. Variance of Physical Activity Decline Rate Explained by Different Blocks of Covariates in Participants Who Did Not Use Walking Aid During Testing (n=615) eTable 7. Variance of Declining Daily Physical Activity Explained by Different Blocks of Covariates in Participants Who Did Not Have a Parkinson’s Disease Diagnosis (n=629) eTable 8. Variance of Declining Daily Physical Activity Explained by Different Blocks of Covariates in Participants Who Did Not Die During the Follow Up (n=408) eTable 9. Use of the Slope of Declining Daily Physical Activity Estimated by 650 Separate Linear Regression Models for Identification of the Variance of Declining Daily Physical Activity Explained by Different Blocks of Covariates eTable 10. Use of the Change in the Level of Physical Activity Between Baseline and the First Follow Up Divided by the Visits’ Time Interval as the Outcome in Relation to the 12 Blocks of Variables eTable 11. Associations of Baseline Turn Speed and Hand Dexterity With the Level of Physical Activity at Years 2, 4, and 6 in 157 Participants Who Had 6+ Measurements of Physical Activity [file jamanetwopen-e2432033-s001.pdf]

Supplemental Online Content

Oveisgharan S, Wang T, Hausdorff JM, Bennett DA, Buchman AS. Motor and nonmotor measures and declining daily physical activity in older adults. *JAMA Netw Open*. 2024;7(9):e2432033. doi:10.1001/jamanetworkopen.2024.32033

eFigure. Flow Chart of Sample Selection From the Rush Memory and Aging Project (MAP) Participants

eTable 1. Comparison of Included vs. Excluded Participants

eTable 2. The 17 Summary Metrics Derived From the Waist Sensor Recordings Quantifying Three Performances: A 32 ft Walk, a Timed Up and Go (TUG) Task, and Standing Balance Tasks

eMethods

eTable 3. A Linear Mixed Effects Model to Estimate Person-Specific Slope of Change of Daily Physical Activity

eTable 4. Associations of Sensor-Derived Walking Metrics With the Rate of Physical Activity Decline in Participants With Valid Manually Calculated Gait Speed (n=323)

eTable 5. Variance of Physical Activity Decline Rate Explained by Different Blocks of Covariates in Participants Without Dementia (n=630)

eTable 6. Variance of Physical Activity Decline Rate Explained by Different Blocks of Covariates in Participants Who Did Not Use Walking Aid During Testing (n=615)

eTable 7. Variance of Declining Daily Physical Activity Explained by Different Blocks of Covariates in Participants Who Did Not Have a Parkinson's Disease Diagnosis (n=629)

eTable 8. Variance of Declining Daily Physical Activity Explained by Different Blocks of Covariates in Participants Who Did Not Die During the Follow Up (n=408)

eTable 9. Use of the Slope of Declining Daily Physical Activity Estimated by 650 Separate Linear Regression Models for Identification of the Variance of Declining Daily Physical Activity Explained by Different Blocks of Covariates

eTable 10. Use of the Change in the Level of Physical Activity Between Baseline and the First Follow Up Divided by the Visits' Time Interval as the Outcome in Relation to the 12 Blocks of Variables

© 2024 Oveisgharan S et al. *JAMA Network Open*

eTable 11. Associations of Baseline Turn Speed and Hand Dexterity With the Level of Physical Activity at Years 2, 4, and 6 in 157 Participants Who Had 6+ Measurements of Physical Activity

This supplemental material has been provided by the authors to give readers additional information about their work.

© 2024 Oveisgharan S et al. *JAMA Network Open*

eFigure. Flow Chart of Sample Selection From the Rush Memory and Aging Project (MAP) Participants

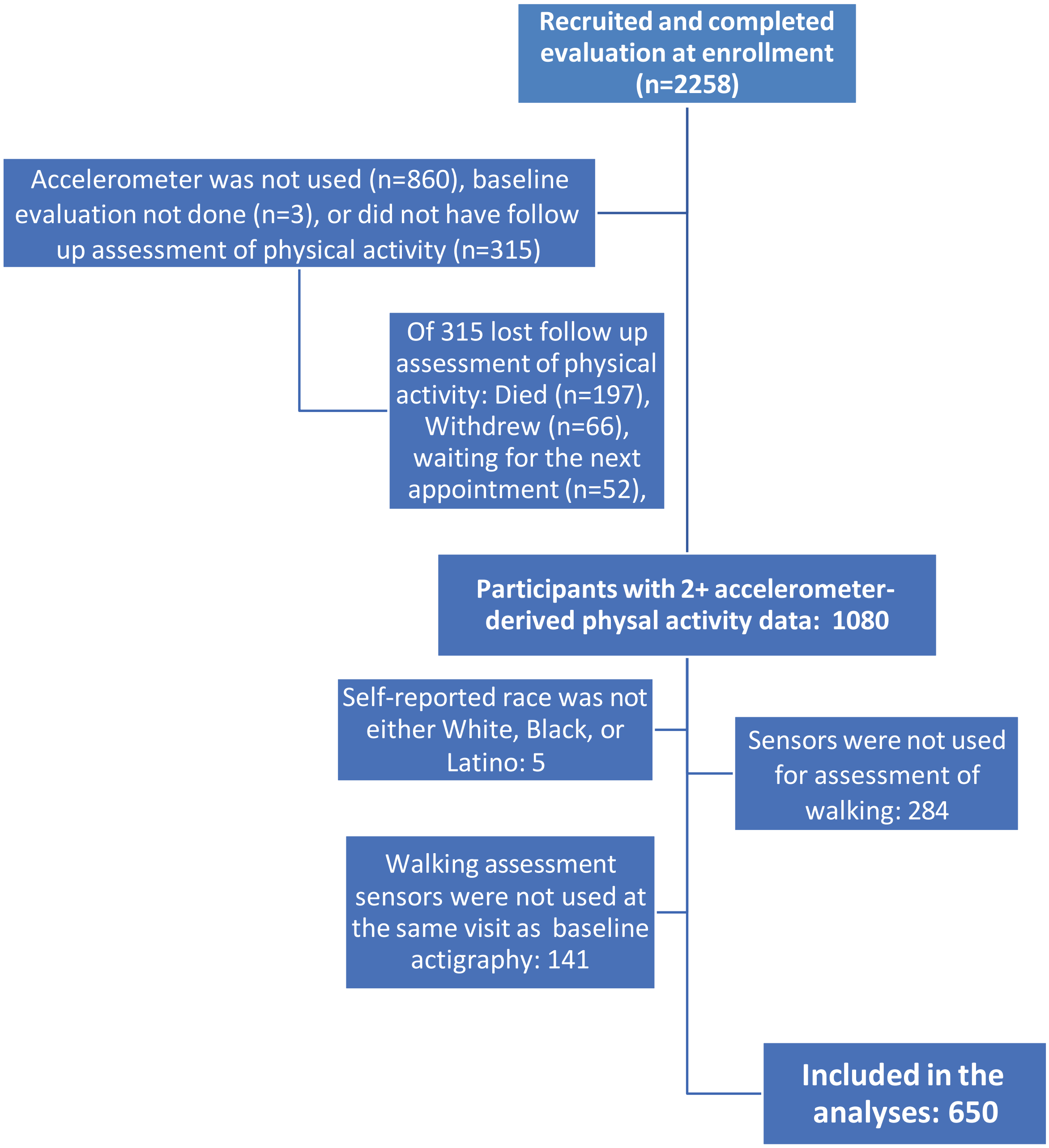

© 2024 Oveisgharan S et al. *JAMA Network Open*

eTable 1. Comparison of Included vs. Excluded Participants

Characteristics at enrollment	Included (n=650)	Excluded (n=1608)	Estimate	P-value
**Age**	78.4 (7.4)	80.7 (7.6)	t=-6.6	<0.001
**Sex**			χ^2=6.0	0.01
**Women, n (%)**	500 (76.9)	1156 (71.9)		
**Men, n (%)**	150 (23.1)	452 (28.1)		
**Education**	15.6 (3.0)	14.7 (3.5)	t=6.4	<0.001
**Race/Ethnicity**			χ^2=22.3	<0.001
**Latino, n (%)**	17 (2.6)	104 (6.5)		
**Non-Latino Black, n (%)**	31 (4.8)	84 (5.2)		
**Non-Latino White, n (%)**	602 (92.6)	1401 (87.1)		
**Other (American Indian or Alaska Native, Asian, Native Hawaiian or other Pacific Islander) , n (%)**	0	19 (1.2)		
**Self-report physical exercise (hours/week)**	3.88 (3.65)	3.19 (3.70)	KW χ^2=41.4	<0.001
**Self-report cognitive activity (1-5)**	3.27 (0.62)	3.15 (0.74)	t=3.9	<0.001
**Self-report social activity (1-5)**	2.76 (0.53)	2.54 (0.59)	t=8.8	<0.001
**Disability in instrumental activity of daily living (0-8)**	0.58 (1.06)	1.22 (1.75)	KW χ^2=68.7	<0.001
**Disability in basic activities of daily living (0-6)**	0.08 (0.38)	0.28 (0.83)	KW χ^2=38.4	<0.001
**Mobility disability (0-3)**	0.49 (0.80)	0.83 (1.05)	KW χ^2=46.2	<0.001
**Dementia, n (%)**	9 (1.4)	112 (7.0)	χ^2=28.5	<0.001

Characteristics of the participants were compared using t-test, chi-square, and Kruskal-Wallis tests.

© 2024 Oveisgharan S et al. *JAMA Network Open*

eTable 2. The 17 Summary Metrics Derived From the Waist Sensor Recordings Quantifying Three Performances: A 32 ft Walk, a Timed Up and Go (TUG) Task, and Standing Balance Tasks

Subtask	Composite summary metrics	Measure[a]	Description
**32ft-Walk**	**Regularity**	Stride regularity	Regularity is the autocorrelation of the vertical acceleration signal time series. Therefore, values closer to 1 indicate that in an individual stronger correlation was observed between the recorded signals across walking and, hence, more similar steps.
	**Cadence**	Cadence (steps/min)	Number of steps/min
	**Pace**	Walking speed (ft/s)	Distance/time to complete subtask
		Step length (ft)	Distance/number of steps
	**Variability**	Step time CV (%)	COV of step time (std/mean step time x 100)
**TUG Sit to stand**	**Complexity**	Pitch SD (deg/s)	Standard deviation of pitch (mediolateral [ML] rotation)
		Pitch jerk (deg/s^2)	Slope during the transition interval (ML axis)
		AP acceleration SD (ft/s^2)	Standard deviation of anterior-posterior (AP) axis acceleration
		-1*AP jerk (ft/s^3)	Slope during the transition interval (AP axis)
	**Duration**	Pitch duration (s)	Duration of transition interval: ML axis
		AP duration (s)	Duration of transition interval: AP axis
**TUG Stand to sit**	**Descent control**	Pitch SD (deg/s)	Standard deviation of pitch
		AP acceleration SD (ft/s^2)	Standard deviation of AP axis acceleration
	**Smoothness**	Pitch jerk (deg/s^2)	Slope during the transition interval (ML axis)
		-1*Pitch duration (s)	Duration of transition interval: ML axis
		AP jerk (ft/s^3)	Slope during the transition interval (AP)
		-1*AP duration (s)	Duration of transition interval: AP axis
**TUG Turn**	**Speed**	Yaw Amp(deg/s)	Amplitude of angular velocity (rotation) around the vertical axis
	**Duration of first turn**	Duration (s)	Duration of the first turn, which is between walking forth and back
	**Duration of second turn**	Duration (s)	Duration of the second turn, which is between walking back and sitting down
**Standing with open eyes**	**Sway magnitude**	Average velocity (ft/s)	Average velocity of sway around Transverse Plane (AP and ML axes)
		Total power	Total power of the recordings calculated in the frequency band 0.15 to 3.5 Hz
		Acceleration (ft/s^2)	Acceleration of sway around Transverse Plane
	**Sway frequency**	Centroid Frequency (1/s)	Zero crossing frequency
	**Sway Jerk**	Sway Jerk (mm/s^3)	Slope of acceleration in the Transverse Plane

© 2024 Oveisgharan S et al. *JAMA Network Open*

Subtask	Composite summary metrics	Measure[a]	Description
**Standing with closed eyes**	**Sway magnitude**	Average velocity (ft/s)	Average velocity of sway around Transverse Plane
		Total power	Total power of the recordings calculated in the frequency band 0.15 to 3.5 Hz
		Acceleration (ft/s^2)	Acceleration of sway around Transverse Plane
	**Sway frequency**	Centroid Frequency (1/s)	Zero crossing frequency
	**Sway Jerk**	Sway Jerk (mm/s^3)	Slope of acceleration in the Transverse Plane

[a]All the measures were log_{10} transformed and standardized.

© 2024 Oveisgharan S et al. *JAMA Network Open*

eMethods.

Use of a wrist sensor to quantify physical activity

Details of the methods of quantifying daily physical activity using wrist sensors have been published before[1,2]. Each participant was fitted with a watch-like omnidirectional accelerometer device (Actical®; Mini Mitter, Bend, OR) on the non-dominant wrist and was requested to wear it continuously for at least seven and up to ten days until a return visit from MAP study staff to collect the device. We excluded from the analyses any 24-hour period during which the Actical removal was confirmed or suspected of not being worn (long bouts of zero activity). We analyzed the data for all available complete days for each participant (range 7-10) in order to provide the highest possible signal-to-noise ratio in the total daily physical activity metric. We required a minimum of seven complete days to be included in the current study and did not differentiate between weekday and weekend days.

Actical contains an omnidirectional piezoelectric accelerometer whose analog output signal is amplified and bandpass-filtered (0.5 and 3.0 Hz cutoff frequencies) before being digitized at a sampling rate of 32 Hz, rectified via comparison with the baseline level of sensed acceleration (e.g. gravity), and summed over each 15-second epoch by an onboard microprocessor to yield an activity count for the epoch, which is stored in the device's memory until data download to a computer. We summed the activity counts of 15s epochs for each 24-hour recordings to yield daily physical activity counts, which were averaged across days to make physical activity counts/day that was used as the primary measure analyzed in this study.

Use of a waist sensor to quantify mobility metrics

Details of the methods of quantifying daily physical activity using wrist sensors have been published before[2,3]. During the annual structured motor exam, MAP participants were asked to complete select mobility performances while wearing a body-fixed sensor (DynaPort® Hybrid, McRoberts B.V., Netherlands). Participants were asked if they understood the instructions before beginning each performance. Practice trials were not offered. The mobility performances included a) walk 32 feet, b) Timed Up and Go (TUG), and c) standing balance for 20 seconds.

The waist sensor consists of three orthogonally oriented piezoelectric accelerometers and three gyroscopic sensors for 100-Hz sampling of three-dimensional acceleration and rotation rate of the lower trunk, the body segment to which the device is fixed via its positioning on a belt around the waist. The DynaPort data acquisition, processing, and analysis stream has been designed and implemented at the Rush Alzheimer's Disease Center to accommodate annual instrumented gait testing harmonized across several large cohorts. This stream proceeds according to the following steps:

1) The pipeline begins with structured mobility testing in the community setting. For the full, unmodified battery of mobility performances, a floor space with a long dimension of at least 8 feet is used, although the protocol can be abbreviated or modified to accommodate smaller spaces. Prior to testing, a research assistant (RA) formats the flash storage aboard the DynaPort device and enters the participant's unique 8-digit ID. The device is then fastened upon the lower back by means of an elastic Velcro belt. During the course of the performances, digital "markers" are placed within the continuously recorded data to denote the beginning and end of each performance (e.g. walking, TUG, standing posture with eyes open and closed), since these time points can be difficult or impossible to ascertain by referencing the acceleration and rotation rate signals alone. Insertion of the digital markers is triggered via wireless Bluetooth signals sent from a nearby notebook computer, which in turn is controlled remotely by means of a wireless, handheld "clicker" device. Thus, the RA is free to be at arm's reach of the participant at all times, should that individual require assistance during any of the performances.

© 2024 Oveisgharan S et al. *JAMA Network Open*

2) At the conclusion of each participant's structured mobility testing session, the DynaPort is connected to the encrypted notebook computer via USB. The acquired data is automatically transferred to a reserved directory on the computer and subsequently removed from the DynaPort device in preparation for the next participant's test session. Once a reliable Internet connection is available, data are uploaded to a central server with RAID and offsite backup storage schemes guarding against loss. The central server matches uploaded data files to the appropriate participant ID and annual visit number based on a comparison of metadata in the file to the list of scheduled testing sessions in the system. The absence of an expected mobility testing file on the central server 24 hours after the scheduled testing time triggers an automated e-mail reminder alerting the data coordinator to the missing data, facilitating timely follow-up with the RA who collected the data.

3) Quality control (QC) procedures are most efficiently employed on batches of approximately 50 gait files at a time. An RA specialized in data processing uses a custom graphic user interface (GUI) developed in Matlab to view the entirety of the acceleration and rotation rate data, identifying segments corresponding to specific performances by referencing both the digital markers within the file as well as the collecting RA's record of the participant's refusal or inability to perform portions of the test and other protocol deviations. The time to complete this initial QC and annotation of the data is on average 2.2 minutes per file (SD = 2.2). There was complete intra-rater and inter-rater agreement (n=608 and n=100, respectively) in identification of the appropriate sections of data corresponding to the three mobility performances, reflections of both the streamlined approach facilitated by the GUI and the inherent quality of the collected data. The data for each individual performance is written to a separate text file in preparation for subsequent processing steps.

4) The isolated data for each performance are fed into performance-specific GUIs that allow a secondary visual QC check. Depending on the performance being processed, the GUI may also prompt the trained operator to, for example, identify a peak in the vertical acceleration curve corresponding to a specific feature of the performance. Scalar gait and balance parameters are then calculated using previously described formulas[4–6] and checked for values outside of the expected range. The presence of outliers triggers another repetition of the QC, performance segmentation, and calculation of gait and balance parameters for those cases in order to understand and correct (if possible) the source of any errors.

Gait and balance parameters extracted in the previous step are written to unique text files for each participant, visit number, and mobility performance. These text files are uploaded to the central server, where they are automatically matched to the appropriate participant and visit number, facilitating their integration in analyses with other clinical measures.

Nine blocks including 41 covariates examined together with sensor-derived mobility metrics in relation to declining daily physical activity.

1- Demographics. Date of birth, sex, race, ethnicity, and years of education were obtained at MAP study entry interview through self-report questions. Age at analytic baseline was calculated using date of visit and date of birth. Participants were asked to identify their race from the following options: American Indian or Alaska Native, Asian, Native Hawaiian or Other Pacific Islander, Black or African-American, White, and Other (to be named). In addition, participants were asked to choose yes/no in response to these questions: "Are you of Spanish, Hispanic or Latino origin? That is, having origins from a Spanish-speaking or Latin American country?" From the response to the race/ethnicity question, participants were classified into Black, Latino, White, and other. Black was ascribed if the race was "Black or African-

American" and the ethnicity was not Latino, Latino if the ethnicity was "Latino", White if the race was "White" and ethnicity was not Latino, and other if race was neither Black nor White, and ethnicity was not Latino.

2- Other wrist sensor-derived metrics.Several other previously published metrics were extracted from the continuous multiday wrist-worn sensor recordings. Two metrics were derived describing day-to-day similarity of the rest-activity pattern (***interdaily stability***) and a measure of the magnitude of hour-to-hour fluctuations of activity counts (***intradaily variability***)[7].

Fractal metrics, designated as α, describe temporal correlations of fluctuations in physical activity. A values greater than 0.5 indicates positive correlations where higher levels of physical activity are followed by higher levels and vice versa, and α values less than 0.5 indicates negative correlations. Two ***fractal metrics*** were calculated, describing correlations in the fluctuations of physical activity in two time scales: recordings of physical activity between 1.5 minutes to 90 minutes, and from 2 to 10 hours[8].

Lastly, we examined a metric describing the probability that an epoch showing no recorded activity counts (rest) was likely to be followed by an epoch of non-zero activity. This measure is a marker of ***sleep fragmentation*** with higher values indicating more fragmented rest/sleep[7].

3- Medical conditions and vascular risk factors. Composite variables indicating number of vascular risk factors (hypertension, diabetes mellitus, and smoking) and vascular diseases (stroke, myocardial infarction, congestive heart failure, lower extremities claudication) were created from self-report medical history. Body mass index (BMI) was calculated using measured weight and height.

4- Cognition. Annually, 19 neuropsychological tests were administered for assessment of 5 cognitive domains as described previously[9,10]. Of the 19 tests, 7 tests were used for assessment of episodic memory, 4 tests for processing speed, 3 tests for semantic memory, 3 tests for working memory, and 2 tests for visuospatial ability. Participants' scores were standardized, using MAP baseline scores, and were averaged to create the 5 cognitive domains' scores.

Scores of the neuropsychological tests were rated by an algorithm and reviewed by a neuropsychologist. The neuropsychologist's cognitive assessment and other health data including neurological examinations were reviewed by a dementia expert to adjudicate cognitive status, of no cognitive impairment (NCI), mild cognitive impairment (MCI) or dementia based on published criteria[11,12]. Baseline cognitive status was used only to describe the analytic cohort or for sensitivity analyses and was not used as a covariate to avoid duplicating cognitive testing scores.

5- Psychosocial. Depressive symptoms were assessed using a short, 10-item, version of Center for Epidemiologic Studies Depression scale[13,14]. Purpose in life was assessed using a 10-item version of Ryff's scales of Psychological Well-Being. Participants responded to the items on a 5-point rating scale, which were averaged across the 10 items[15]. Social network size was the number of children, family, and friends that each participant interacted with at least once in a month[16]. Loneliness was assessed using a 5-item version of the de Jong-Gierveld Loneliness Scale on a 5-point rating scale[17]. Well-being was assessed with an 18-item version of Ryff's Scales of Psychological Well Being on a 7-point rating scale[18].

6- Pulmonary function. Pulmonary function was assessed using a hand-held spirometer [MicroPlus Spirometer MS03, MicroMedical Ltd. Kent, UK]. This device provided several measures of function including: forced vital capacity, forced expiratory volume in one second, and peak expiratory flow rate. Two trials of each measure were collected from each participant and were averaged [19].

© 2024 Oveisgharan S et al. *JAMA Network Open*

7- Self-report activities and disabilities. Self-report questions were used to obtain information about varied late-life physical, cognitive and social activities and disabilities. Participants reported the time spent in 5 physical activities, which was summarized as hours of activity per week[20]. Cognitive activity was assessed by seven questions asking about participation in seven cognitive activities, including reading books and playing games, using a 1-5 rating scale[21]. Similarly, six questions asked about participation in six social activities including going to restaurants and visiting friends and families[22].

Three scales were used to assess disability. Disability in instrumental activities of daily living (IADL) was measured using eight items adapted from the Duke Older Americans Resources and Services project. The composite measure ranged from 0-8 and represented number of items for which participants needed help or were unable to accomplish the activity[23]. Similarly, participants were asked about needing help or disability in doing six basic physical activities (ADL) [23] or mobility disability based on 3 activities including walking around the house, walking up and down stairs, and walking one half a mile[24].

8- Non-sensor conventional motor assessment. Several additional motor tasks and assessments were employed to capture varied facets of motor function in aging adults. Participants were asked to tap an electronic tapper (Western Psychological Services, Los Angeles, CA) with index finger of each hand twice as fast as they could in 10 seconds. Separately, in the Purdue Pegboard test participants were asked to correctly insert pegs into the pegboard in 30 seconds. Each of the two tasks were repeated four times, twice with each hand, and the scores of the four trials were averaged and subsequently divided by sex-specific means, which were averaged to yield a composite hand dexterity score[25]. *Hand strength* was a composite measure derived from assessments of grip and pinch strength using a hand-held and pinch dynamometer[25].

Parkinsonism: Four parkinsonian signs were rated by trained nurses using a modified motor portion of the Unified Parkinson’s Disease Rating Scale. These four signs were summarized by a global parkinsonism severity score ranging from 0-100[26]. The parkinsonism score was square root transformed to make its distribution closer to normal as done in previous studies[26].

9- Laboratory measures. Blood was annually drawn and analyzed by Quest Diagnostics for levels of the following: hemoglobin A_1C, triglyceride, high-density lipoprotein cholesterol, thyroid stimulating hormone, hemoglobin, and creatinine were measured. Levels of low-density lipoprotein cholesterol and glomerular filtration rate were estimated[27–30].

Statistical analysis

Using linear mixed effect models with fixed and random terms for intercept (baseline level of physical activity) and Time (annual rate of change in physical activity), we estimated person-specific slope of change in daily physical activity. It was further used as the outcome in subsequent analyses. We transformed physical activity counts by a natural logarithmic transformation to meet assumptions of linear mixed effects models (eMethods)[31,32].

In the next stage of analyses we examined the associations of variables, classified in 12 blocks, with the person-specific slope of change in daily physical activity, described above, as a measure of longitudinal change of physical activity. In 12 linear regression models, we examined each block of variables separately in relation to the slope of change of daily physical activity. Based on this two-stage analysis, we can use the adjusted r^2 for each of the 12 linear regression models to quantify the percent of variance in the slope of change of daily physical activity explained by each of the 12 blocks of variables. We used bootstrap with 1000 repetitions to obtain 95% confidence interval of the adjusted r^2. In a final

© 2024 Oveisgharan S et al. *JAMA Network Open*

model, we examined in a single model all the variables that were significantly related to the slope of change of daily physical activity in the individual blocks of variables.

In a sensitivity analysis, we replaced the slope of declining physical activity estimated using a linear mixed effect model with the slopes estimated from 650 separate linear regression models where the outcome was each participant's longitudinal measurements of daily physical activity in a separate model.

To address multicollinearity, we examined variables with variance inflation factor greater than 10 in separate models. To address multiple comparisons, we applied Benjamini and Hochberg false discovery rate (FDR)[33], and significant variables were the ones with FDR-corrected p-value (q-value) less than 0.05. The FDR was applied separately in each block including the 3 blocks of sensor-derived metrics of the three motor performances. The analyses were done using SAS version 15.2 (SAS Institute, Cary, NC).

The rationale for using logarithmic transformation of daily physical activity counts

We found that we were unable to examine raw daily physical activity measures in a linear mixed effects model due to the high correlation between the estimated baseline level and slope of activity decline (Spearman $\rho = -0.76$). This correlation indicated that those who had a higher baseline level of physical activity had a faster decline (more negative slope) of physical activity compared with those with a lower baseline level of physical activity. This is illustrated in the figure below in the trajectories of raw daily physical activity in those with high ($\geq 90^{th}$ percentile) vs. low ($\leq 10^{th}$ percentile) level of baseline physical activity.

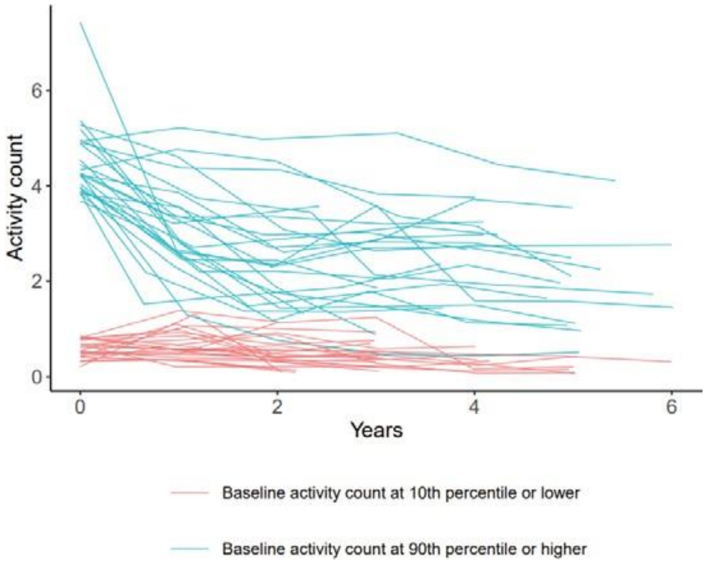

The negative association between the baseline physical activity level and its rate of decline indicates that the longitudinal physical activity change probably is a proportional change. That motivated us to consider a logarithmic transformation of the data which could transform a proportional change to a magnitude change as assumed in typical linear mixed-effects models. When we examined the log-transformed physical activity in a linear mixed-effects model, the correlation between the estimated baseline level and the slope of change of the log-transformed level of physical activity was low (Spearman $\rho=0.15$), suggesting that the slope of decline in the log-transformed level of physical activity was not driven by the baseline level of physical activity. This characteristic provided us more power to identify correlates of the rate of decline in physical activity, and hence, for our analyses we chose log-transformed daily physical activity as our primary outcome measure.

© 2024 Oveisgharan S et al. *JAMA Network Open*

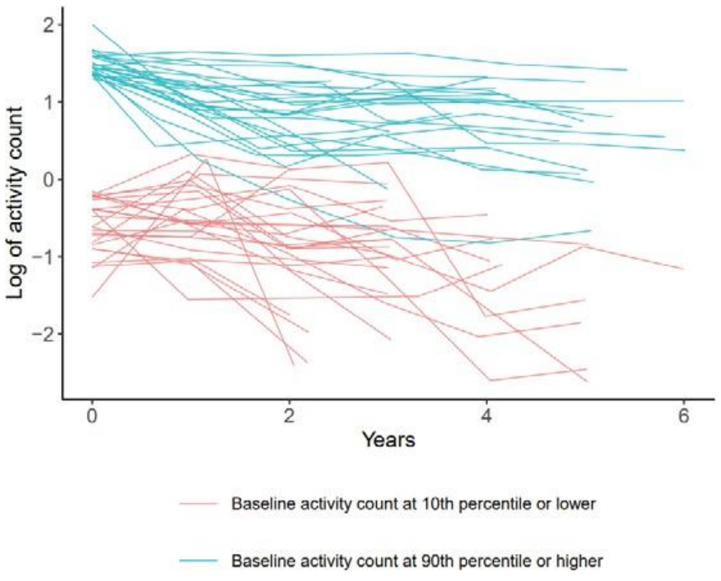

eReferences

1. Buchman AS, Boyle PA, Yu L, Shah RC, Wilson RS, Bennett DA. Total daily physical activity and the risk of AD and cognitive decline in older adults. *Neurology*. 2012;78(17):1323-1329. doi:10.1212/WNL.0b013e3182535d35

2. Dawe RJ, Leurgans SE, Yang J, et al. Association Between Quantitative Gait and Balance Measures and Total Daily Physical Activity in Community-Dwelling Older Adults. *The journals of gerontology Series A, Biological sciences and medical sciences*. 2018;73(5):636-642. doi:10.1093/gerona/glx167

3. Buchman AS, Dawe RJ, Leurgans SE, et al. Different combinations of mobility metrics derived from a wearable sensor are associated with distinct health outcomes in older adults. *J Gerontol A Biol Sci Med Sci*. 2020;75(6):1176-1183. doi:10.1093/gerona/glz160

4. Matinolli M, Korpelainen JT, Korpelainen R, Sotaniemi KA, Virranniemi M, Myllylä VV. Postural sway and falls in Parkinson’s disease: a regression approach. *Mov Disord*. 2007;22(13):1927-1935. doi:10.1002/mds.21633

5. Weiss A, Herman T, Plotnik M, Brozgol M, Giladi N, Hausdorff JM. An instrumented timed up and go: the added value of an accelerometer for identifying fall risk in idiopathic fallers. *Physiol Meas*. 2011;32(12):2003-2018. doi:10.1088/0967-3334/32/12/009

© 2024 Oveisgharan S et al. *JAMA Network Open*

6. Weiss A, Herman T, Plotnik M, et al. Can an accelerometer enhance the utility of the Timed Up & Go Test when evaluating patients with Parkinson's disease? *Med Eng Phys*. 2010;32(2):119-125. doi:10.1016/j.medengphy.2009.10.015

7. Lim ASP, Yu L, Costa MD, et al. Quantification of the fragmentation of rest-activity patterns in elderly individuals using a state transition analysis. *Sleep*. 2011;34(11):1569-1581. doi:10.5665/sleep.1400

8. Li P, Yu L, Yang J, et al. Interaction between the progression of Alzheimer's disease and fractal degradation. *Neurobiol Aging*. 2019;83:21-30. doi:10.1016/j.neurobiolaging.2019.08.023

9. Oveisgharan S, Dawe RJ, Yu L, et al. Frequency and underlying pathology of pure vascular cognitive impairment. *JAMA Neurol*. 2022;79(12):1277-1286. doi:10.1001/jamaneurol.2022.3472

10. Oveisgharan S, Wilson RS, Yu L, Schneider JA, Bennett DA. Association of Early-Life Cognitive Enrichment With Alzheimer Disease Pathological Changes and Cognitive Decline. *JAMA Neurol*. 2020;77(10):1217. doi:10.1001/jamaneurol.2020.1941

11. McKhann GM, Knopman DS, Chertkow H, et al. The diagnosis of dementia due to Alzheimer's disease: recommendations from the National Institute on Aging-Alzheimer's Association workgroups on diagnostic guidelines for Alzheimer's disease. *Alzheimers Dement*. 2011;7(3):263-269. doi:10.1016/j.jalz.2011.03.005

12. Albert MS, DeKosky ST, Dickson D, et al. The diagnosis of mild cognitive impairment due to Alzheimer's disease: recommendations from the National Institute on Aging-Alzheimer's Association workgroups on diagnostic guidelines for Alzheimer's disease. *Alzheimers Dement*. 2011;7(3):270-279. doi:10.1016/j.jalz.2011.03.008

13. Wilson RS, Capuano AW, Boyle PA, et al. Clinical-pathologic study of depressive symptoms and cognitive decline in old age. *Neurology*. 2014;83(8):702-709. doi:10.1212/WNL.0000000000000715

14. Fleischman DA, Arfanakis K, Leurgans SE, et al. Late-life depressive symptoms and white matter structural integrity within older Black adults. *Front Aging Neurosci*. 2023;15:1138568. doi:10.3389/fnagi.2023.1138568

15. Boyle PA, Wang T, Yu L, Barnes LL, Wilson RS, Bennett DA. Purpose in Life May Delay Adverse Health Outcomes in Old Age. *Am J Geriatr Psychiatry*. 2022;30(2):174-181. doi:10.1016/j.jagp.2021.05.007

16. Bennett DA, Schneider JA, Tang Y, Arnold SE, Wilson RS. The effect of social networks on the relation between Alzheimer's disease pathology and level of cognitive function in old people: a longitudinal cohort study. *Lancet Neurol*. 2006;5(5):406-412. doi:10.1016/S1474-4422(06)70417-3

17. Buchman AS, Boyle PA, Wilson RS, et al. Loneliness and the rate of motor decline in old age: the Rush Memory and Aging Project, a community-based cohort study. *BMC Geriatr*. 2010;10:77. doi:10.1186/1471-2318-10-77

18. Wilson RS, Boyle PA, Segawa E, et al. The influence of cognitive decline on well-being in old age. *Psychol Aging*. 2013;28(2):304-313. doi:10.1037/a0031196

19. Buchman AS, Boyle PA, Wilson RS, Gu L, Bienias JL, Bennett DA. Pulmonary function, muscle strength and mortality in old age. *Mech Ageing Dev*. 2008;129(11):625-631. doi:10.1016/j.mad.2008.07.003

20. Buchman AS, Boyle PA, Wilson RS, Bienias JL, Bennett DA. Physical activity and motor decline in older persons. *Muscle Nerve*. 2007;35(3):354-362. doi:10.1002/mus.20702

21. Wilson RS, Segawa E, Boyle PA, Bennett DA. Influence of late-life cognitive activity on cognitive health. *Neurology*. 2012;78(15):1123-1129. doi:10.1212/WNL.0b013e31824f8c03

22. Buchman AS, Boyle PA, Wilson RS, Fleischman DA, Leurgans S, Bennett DA. Association between late-life social activity and motor decline in older adults. *Arch Intern Med*. 2009;169(12):1139-1146. doi:10.1001/archinternmed.2009.135

23. Boyle PA, Buchman AS, Wilson RS, Bienias JL, Bennett DA. Physical activity is associated with incident disability in community-based older persons. *J Am Geriatr Soc*. 2007;55(2):195-201. doi:10.1111/j.1532-5415.2007.01038.x

24. Buchman AS, Boyle PA, Leurgans SE, Evans DA, Bennett DA. Pulmonary Function, Muscle Strength, and Incident Mobility Disability in Elders. *Proceedings of the American Thoracic Society*. 2009;6(7):581-587. doi:10.1513/pats.200905-030RM

25. Buchman AS, Wilson RS, Leurgans SE, Bennett DA, Barnes LL. Change in motor function and adverse health outcomes in older African-Americans. *Exp Gerontol*. 2015;70:71-77. doi:10.1016/j.exger.2015.07.009

26. Oveisgharan S, Yu L, Dawe RJ, Bennett DA, Buchman AS. Total daily physical activity and the risk of parkinsonism in community-dwelling older adults. *The journals of gerontology Series A, Biological sciences and medical sciences*. 2019;75(4):702-711.

27. Oveisgharan S, Capuano AW, Nag S, et al. Association of hemoglobin A1C with TDP-43 pathology in community-based elders. *Neurology*. 2021;96(22):e2694-e2703. doi:10.1212/WNL.0000000000012025

28. Oveisgharan S, Kim N, Agrawal S, et al. Brain and spinal cord arteriolosclerosis and its associations with cerebrovascular disease risk factors in community-dwelling older adults. *Acta Neuropathol*. 2023;145(2):219-233. doi:10.1007/s00401-022-02527-z

29. Shah RC, Buchman AS, Wilson RS, Leurgans SE, Bennett DA. Hemoglobin level in older persons and incident Alzheimer disease: prospective cohort analysis. *Neurology*. 2011;77(3):219-226. doi:10.1212/WNL.0b013e318225aaa9

30. Buchman AS, Tanne D, Boyle PA, Shah RC, Leurgans SE, Bennett DA. Kidney function is associated with the rate of cognitive decline in the elderly. *Neurology*. 2009;73(12):920-927. doi:10.1212/WNL.0b013e3181b72629

© 2024 Oveisgharan S et al. *JAMA Network Open*

31. Buchman AS, Wang T, Oveisgharan S, et al. Correlates of Person-Specific Rates of Change in Sensor-Derived Physical Activity Metrics of Daily Living in the Rush Memory and Aging Project. *Sensors (Basel)*. 2023;23(8):4152. doi:10.3390/s23084152

32. Buchman AS, Wang T, Oveisgharan S, et al. Correlated decline of cognitive and motor phenotypes and ADRD pathologies in old age. *Alzheimers Dement*. Published online June 12, 2023. doi:10.1002/alz.13347

33. Benjamini Y, Hochberg Y. Controlling the false discovery rate: A practical and powerful approach to multiple testing. *Journal of the Royal Statistical Society: Series B (Methodological)*. 1995;57(1):289-300. doi:10.1111/j.2517-6161.1995.tb02031.x

© 2024 Oveisgharan S et al. *JAMA Network Open*

eTable 3. A Linear Mixed Effects Model to Estimate Person-Specific Slope of Change of Daily Physical Activity

Model term	Estimate (SE)	P-value
**Intercept**	0.577 (0.022)	<0.001
**Time**	-0.184 (0.007)	<0.001

The cells' numbers are derived from a linear mixed effects model. The outcome was longitudinal measures of logarithmic transformation of daily activity counts and the fixed and random model terms were intercept and time.

© 2024 Oveisgharan S et al. *JAMA Network Open*

eTable 4. Associations of Sensor-Derived Walking Metrics With the Rate of Physical Activity Decline in Participants With Valid Manually Calculated Gait Speed (n=323)

**Covariates**	**Association with daily physical activity decline rate**	
	**Estimate (SE)**	**P-value**
**Sensor-derived walking metrics**		
Regularity	0.022 (0.007)	0.002
Cadence (steps/min)	0.004 (0.005)	0.48
Pace	0.027 (0.005)	<0.001
Variability of step time	0.005 (0.005)	0.48
Variance explained by the covariates = 22.9% (13.1%–34.1%)		

© 2024 Oveisgharan S et al. *JAMA Network Open*

eTable 5. Variance of Physical Activity Decline Rate Explained by Different Blocks of Covariates in Participants Without Dementia (n=630)

Blocks of covariates	Variance of physical activity decline rate explained by blocks of covariates
**Demographic**	14.5% (10.6%–20.3%)
**Waist sensor-derived mobility metrics-Walking**	21.2% (15.5%–28.0%)
**Waist sensor-derived mobility metrics-Timed Up & GO**	21.5% (16.5%–29.3%)
**Waist sensor-derived mobility metrics-Standing**	2.2% (0.6%–6.8%)
**Wrist sensor-derived other covariates**	11.3% (7.1%–18.1%)
**Vascular factors**	4.4% (1.9%–8.9%)
**Cognition**	9.3% (5.1%–15.7%)
**Psychosocial**	6.8% (3.7%–12.2%)
**Pulmonary function**	5.3% (2.4%–9.3%)
**Self-report activities & disabilities**	17.9% (12.4%–26.5%)
**Conventional motor function**	22.1% (15.8%–30.2%)
**Lab**	6.7% (4.1%–11.7%)

eTable 6. Variance of Physical Activity Decline Rate Explained by Different Blocks of Covariates in Participants Who Did Not Use Walking Aid During Testing (n=615)

**Blocks of covariates**	**Variance of physical activity decline rate explained by blocks of covariates**
**Demographic**	15.7% (11.3%–21.6%)
**Waist sensor-derived mobility metrics-Walking**	21.3% (16.3%–27.8%)
**Waist sensor-derived mobility metrics-Timed Up & GO**	22.2% (17.1%–29.7%)
**Waist sensor-derived mobility metrics-Standing**	3.0% (0.9%–8.1%)
**Wrist sensor-derived other covariates**	9.6% (5.5%–16.4%)
**Vascular factors**	4.3% (1.7%–9.0%)
**Cognition**	8.5% (4.9%–14.7%)
**Psychosocial**	6.1% (3.1%–11.5%)
**Pulmonary function**	5.2% (2.0%–9.5%)
**Self-report activities & disabilities**	18.2% (12.8%–25.6%)
**Conventional motor function**	23.7% (18.3%–29.8%)
**Lab**	7.7% (4.9%–13.6%)

© 2024 Oveisgharan S et al. *JAMA Network Open*

eTable 7. Variance of Declining Daily Physical Activity Explained by Different Blocks of Covariates in Participants Who Did Not Have a Parkinson's Disease Diagnosis (n=629)

Blocks of covariates	Variance of physical activity decline rate explained by blocks of covariates
**Demographic**	14.9% (10.3%–20.3%)
**Waist sensor-derived mobility metrics-Walking**	23.5% (17.8%–30.5%)
**Waist sensor-derived mobility metrics-Timed Up & GO**	22.2% (17.7%–29.5%)
**Waist sensor-derived mobility metrics-Standing**	2.3% (0.6%–7.0%)
**Wrist sensor-derived other covariates**	11.5% (7.2%–17.8%)
**Vascular factors**	4.7% (1.9%–9.3%)
**Cognition**	10.7% (6.6%–17.3%)
**Psychosocial**	7.0% (3.6%–12.8%)
**Pulmonary function**	5.8% (2.4%–10.1%)
**Self-report activities & disabilities**	19.0% (13.4%–27.4%)
**Conventional motor function**	23.5% (17.1%–30.5%)
**Lab**	6.3% (3.8%–11.3%)

© 2024 Oveisgharan S et al. *JAMA Network Open*

eTable 8. Variance of Declining Daily Physical Activity Explained by Different Blocks of Covariates in Participants Who Did Not Die During the Follow Up (n=408)

**Blocks of covariates**	**Variance of physical activity decline rate explained by blocks of covariates**
**Demographic**	20.4% (14.4%–28.7%)
**Waist sensor-derived mobility metrics-Walking**	25.9% (19.0%–35.4%)
**Waist sensor-derived mobility metrics-Timed Up & GO**	27.1% (21.1%–36.6%)
**Waist sensor-derived mobility metrics-Standing**	3.5% (1.0%–11.3%)
**Wrist sensor-derived other covariates**	15.9% (8.9%–26.0%)
**Vascular factors**	7.1% (2.9%–14.1%)
**Cognition**	11.5% (6.2%–19.9%)
**Psychosocial**	8.9% (4.1%–16.9%)
**Pulmonary function**	6.7% (2.4%–13.2%)
**Self-report activities & disabilities**	22.5% (14.6%–33.7%)
**Conventional motor function**	28.9% (21.1%–38.1%)
**Lab**	11.1% (7.6%–18.9%)

© 2024 Oveisgharan S et al. *JAMA Network Open*

eTable 9. Use of the Slope of Declining Daily Physical Activity Estimated by 650 Separate Linear Regression Models for Identification of the Variance of Declining Daily Physical Activity Explained by Different Blocks of Covariates

Blocks of covariates	Variance of physical activity decline rate explained by blocks of covariates
**Demographic**	4.3%
**Waist sensor-derived mobility metrics-Walking**	6.4%
**Waist sensor-derived mobility metrics-Timed Up & GO**	5.9%
**Waist sensor-derived mobility metrics-Standing**	0.8%
**Wrist sensor-derived other covariates**	2.0%
**Vascular factors**	1.9%
**Cognition**	5.5%
**Psychosocial**	1.6%
**Pulmonary function**	1.0%
**Self-report activities & disabilities**	6.2%
**Conventional motor function**	6.0%
**Lab**	1.6%

© 2024 Oveisgharan S et al. *JAMA Network Open*

eTable 10. Use of the Change in the Level of Physical Activity Between Baseline and the First Follow Up Divided by the Visits' Time Interval as the Outcome in Relation to the 12 Blocks of Variables

**Blocks of covariates**	**Variance of physical activity decline rate explained by blocks of covariates**
**Demographic**	0.2%
**Waist sensor-derived mobility metrics-Walking**	1.3%
**Waist sensor-derived mobility metrics-Timed Up & GO**	1.0%
**Waist sensor-derived mobility metrics-Standing**	1.4%
**Wrist sensor-derived other covariates**	2.8%
**Vascular factors**	0%
**Cognition**	1.5%
**Psychosocial**	0%
**Pulmonary function**	0%
**Self-report activities & disabilities**	1.8%
**Conventional motor function**	3.6%
**Lab**	0%

© 2024 Oveisgharan S et al. *JAMA Network Open*

eTable 11. Associations of Baseline Turn Speed and Hand Dexterity With the Level of Physical Activity at Years 2, 4, and 6 in 157 Participants Who Had 6+ Measurements of Physical Activity

**Baseline motor ability**	**Physical activity at Year 2**			**Physical activity at Year 4**			**Physical activity at Year 6**		
	**Estimate (SE)**	**P-value**	**Adjusted R^2**	**Estimate (SE)**	**P-value**	**Adjusted R^2**	**Estimate (SE)**	**P-value**	**Adjusted R^2**
Turn speed	0.050 (0.041)	0.22	0.545	0.189 (0.048)	<0.001	0.573	0.222 (0.059)	<0.001	0.560
Hand dexterity	0.606 (0.265)	0.02		0.420 (0.312)	0.18		1.109 (0.382)	0.004	

In three separate models, the associations of baseline turn speed and hand dexterity were examined with the level of physical activity at the 2nd, 4th, and 6th visits as the outcomes. All three models were controlled for age at baseline, sex, baseline level of physical activity, and the time interval between the baseline and the follow up visits. Of note, the physical activity counts were log-transformed to be congruent with the other analyses.

© 2024 Oveisgharan S et al. *JAMA Network Open*
