# Supplementary material for: Motor and Nonmotor Measures and Declining Daily Physical Activity in Older Adults
Source: JAMA Netw Open. 2024 Sep 5;7(9):e2432033. doi: 10.1001/jamanetworkopen.2024.32033 (PMC11378007; doi:10.1001/jamanetworkopen.2024.32033)
Supplement: Supplement 2. — Data Sharing Statement [file jamanetwopen-e2432033-s002.pdf]

Data Sharing Statement

Oveisgharan. Motor and Nonmotor Measures and Declining Daily Physical Activity in Older Adults. *JAMA Netw Open*. Published September 05, 2024.
doi:10.1001/jamanetworkopen.2024.32033

Data
Data available: Yes
Data types: Deidentified participant data, Data dictionary
How to access data: The data are available and accessible via the Rush Alzheimer's Disease Center Research Resource Sharing Hub at www.radc.rush.edu. To access data, an application should be filled including a brief description of the study premises and a short research plan.
When available: With publication

Supporting Documents
Document types: None

Additional Information
Who can access the data: Researchers whose proposed use of the data has been approved
Types of analyses: For any purpose.
Mechanisms of data availability: After approval of a proposal and a signed data access agreement.
